# Who will you imitate? Studying reciprocal influence in children-robot groups during an imitation game

**DOI:** 10.3389/frobt.2025.1563923

**Published:** 2025-04-01

**Authors:** Giulia Pusceddu, Francesca Cocchella, Michela Bogliolo, Giulia Belgiovine, Linda Lastrico, Francesco Rea, Maura Casadio, Alessandra Sciutti

**Affiliations:** ^1^ COgNiTive Architecture for Collaborative Technologies, Italian Institute of Technology, Genoa, Italy; ^2^ Department of Informatics, Bioengineering, Robotics and Systems Engineering, University of Genoa, Genoa, Italy; ^3^ Scuola di Robotica, Genoa, Italy

**Keywords:** child-robot interaction, group-robot interaction, group dynamics, robot influence, imitation, robots at school

## Abstract

This work aims to advance the understanding of group dynamics in robot-child interactions, focusing on whether, during a motor-imitation task led by a Nao robot, children might be influenced in their action executions by other group members - human or robotic. After testing eighteen groups of four children and teenagers, our findings indicate that participants tend to disregard the robot when it performs atypical gestures, preferring instead to imitate the actions of a human peer. Moreover, we found evidence that, in this scenario, assigning a leadership role to the robot does not, by itself, guarantee compliance from human group members; broader group dynamics must also be taken into account. Further results show that participants are significantly more likely to imitate the robot’s action when the “proactive” group members (i.e., those who initiate actions first) conform to Nao, compared to when they do not. Previous studies suggest that the mutual influence of group members can facilitate interaction with a robotic agent; however, our findings show that the presence of proactive members could also undermine the group’s conformity to the robot. Additionally, these findings highlight the importance of personalizing robots to better integrate into specific group dynamics, enhancing their ability to influence different groups effectively.

## 1 Introduction

Integrating social robots into daily life is one of the ultimate goals for the Human-Robot Interaction (HRI) field, with research focusing, for instance, on robots as caregivers (Sorell and Draper, 2014) or teachers (Belpaeme et al., 2018). In pursuing this goal, researchers cannot ignore the need to provide social robots with the ability to interact with groups of people. Recognizing groups’ most relevant features and studying group dynamics - i.e., the influential interpersonal processes that occur in and between groups over time (Forsyth, 2018) - would provide insights into how to train robots to interact with groups of humans and deal with their members efficiently and naturally. Most of the knowledge in HRI focuses on dyadic interactions. However, in the last decade, non-dyadic HRI publications have increased significantly (Schneiders et al., 2022).

One area of HRI that generates significant interest and could benefit from non-dyadic research is the use of social robots in education (Belpaeme et al., 2018). Understanding the psychological and behavioral mechanisms that come into play during interactions between groups of students and robots would allow to design and deploy robots that are effective and fair as tools or companions for education. Given that students are often children or teenagers, it is crucial to conduct studies that explore group dynamics in these age groups, with a focus on how they interact with and perceive a robot inserted into their team. Additionally, previous social psychology research - e.g., Turner et al. (1994) - emphasizes the relevance of group interaction for many elements of human development, such as the formation of personal identity. Therefore, it is vital to explore these dynamics in groups of young individuals.

**FIGURE 1:**
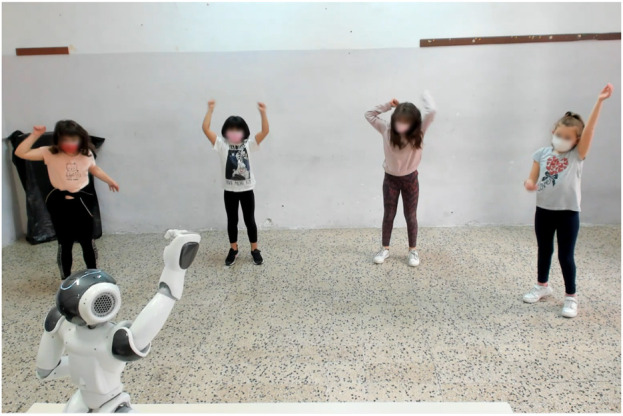
A group of participants mimicking the action “exult” with Nao during the motor imitation game. The side players are imitating the Atypical gesture of the robot, while the central ones are performing the action in the Standard way.

This research can provide valuable insights into the social factors influencing group behavior and can guide the design of robots that foster effective interactions with young individuals. In particular, we investigate how a social robot influences groups of children and teenagers during a motor imitation game. Additionally, we examine how the robot’s impact can be shaped by the social influence exerted by other group members. To do so, we propose a gamified interaction context based on an imitation task (Figure 1 shows a moment of the interaction). During the game, the robot mimed actions in a Typical or Atypical way, thereby conforming or not conforming to the generally acknowledged manner to carry out such actions (see Section 3.3 for more details).

Studying robots’ influence on young people could be relevant to educational settings, where robots might act as tutors or teachers, pushing students to explore new, unfamiliar concepts. By identifying the conditions under which students are more likely to follow a robot’s guidance, even when it introduces ideas students have never encountered before, we can develop adaptive robotic tutors that personalize their approach based on group composition, promoting more effective learning. Another possible application could be in the domain of children’s safety. Understanding how and when children follow robots, even when they exhibit atypical behaviors, could provide insights into their susceptibility. This could be particularly significant, as children and teenagers have been found to be more keen to be influenced than adults (Vollmer et al., 2018; Costanzo and Shaw, 1966). To address these issues, we formulated the following research questions:

**Q1**: Do participants conform to a robot’s atypical behavior during a social game?
**Q2**: Are the participants influenced by the other group members during a social game with a robot?

## 2 Related work

### 2.1 Group dynamics and robots’ influence in HRI

Studying groups is a complex task that cannot rely only on examining individuals separately; indeed, that would mean ignoring fundamental insights such as mutual influences and social contexts (Forsyth, 2018). Previous works have shown some of the challenges and opportunities of investigating human-robot interaction on a group level.

In group **decision-making**, people often rely on implicit social norms, which can lead them to adjust their choices based on others’ input (Sherif, 1936). For instance, Asch (1951) demonstrated that people tend to be influenced by others’ evaluations even when situations are not ambiguous (i.e., evaluation of the length of lines). So far, it is unclear whether the influence of robots in decision-making scenarios within groups is similar to that exerted by humans. Studies using variations of the Asch experiment have found contradictory results Vollmer et al. (2018); Salomons et al. (2018); Qin et al. (2022); Brandstetter et al. (2014). Previous results also suggest that robots can influence group dynamics when they are active participants (Oliveira et al., 2021). Moreover, some studies found that robots can be useful mediators in some conditions. Gillet et al. (2021) observed that a robot’s adaptive gaze behavior could shape the interaction among participants, leading to more participation during a group conversation; others suggest that robots could improve team performance by managing crucial team processes like conflict (Martelaro et al., 2015; Shen et al., 2018).

A factor to consider when integrating robots into human groups is the risk of **group polarization**. As group interaction impacts members by taking their evaluation as individuals to extremes (Moscovici and Zavalloni, 1969), integrating robots into groups could be harder than expected. A recent study found this tendency while investigating polarization in the context of a food-delivery robot: group members were highly influenced by their group mates in evaluating the robot, and they reported a lower trust towards the robot with respect to individual participants (Martinez et al., 2023). These patterns are consistent with other research showing that when people deal with robots, groups - especially cohesive ones - tend to have more negative attitudes toward the robotic devices than individuals (Preusse et al., 2021).

**Group variability** is an additional aspect to take into account. Social psychology research highlights how each member brings to the team a set of unique personal skills, abilities, and motivations, all of which shape how they act as team members. Researchers examined the complex interplay of personality, group composition, and performance by considering the kind of tasks the teams were performing and the personality traits of each member. They found that certain combinations of people, based on their personal motivations and personality traits, are more or less suited to specific types of tasks (Mathieu et al., 2014), which explains why different team compositions may lead to varying outcomes For instance, team members with high emotional stability help the group perform well in conjunctive tasks, but not in tasks that do not rely on coordination among members (Kramer et al., 2014). Another study evidenced that teams composed of all highly dominant individuals are less stable and less productive than groups that include a balance of dominant and less dominant members (Groysberg et al., 2011). Another example is that while a conscientious person fits in well with a team when other members are motivated to perform, that individual may not fit in less task-oriented teams Bell et al. (2015). The combination of different team members working on the same task can, therefore, lead to significantly different outcomes. This has also been observed in recent HRI studies. For instance, Gillet et al. (2022) conducted a participatory design study, asking three groups of teenagers to design and shape the behavior of the Nao robot to mediate and enhance discussion-based activities during a 2-week summer camp. They observed great differences in the designed robot behaviors and perceptions among the teams. Indeed, each team found a different use for the robot, adapting it to the group’s attitudes and identity. A recent study (Axelsson et al., 2023) found that participants’ perceptions of the robot’s current behaviors diverged between the groups tested after a group mindfulness practice in a public cafe. These results together with findings by previous social psychology research suggest that the perception and use of robots may change depending on group members’ attributes and needs, and highlight the necessity for personalized robotics. In the future, efficient and adaptable robots should be able to understand the group with which they interact and tailor their behaviors consequently.

### 2.2 Trust and robots’ influence in child-robot interaction

To design robots that effectively act as tutors or peers in learning tasks, it is important to evaluate their influence on the decision-making of users. Previous research has proven that robots can impact children’s choices and behaviors in social contexts (Stower et al., 2021; Vollmer et al., 2018). However, children’s perception and preferences towards robots seem to evolve rapidly with age (Cocchella et al., 2023; Stower et al., 2021; Sciutti et al., 2014; Kahn Jr et al., 2012), making it challenging to determine the extent of a robot’s influence across different age groups. Trust-related behaviors, for instance, often rely on the development of theory of mind: as children acquire this ability, they begin to evaluate whether an agent is trustworthy based on its informational access (Di Dio et al., 2020). Geiskkovitch et al. (2019) demonstrated that young children tend to trust a robot that has previously provided correct information, aligning with earlier findings in developmental psychology about children’s trust in previously reliable human agents (Birch et al., 2008). Yet in case participants can choose between human and robotic agents, younger children (3 y. o.) display a greater inclination to trust humans over robotic play partners, while older children (7 y. o.) tend to do the opposite (Di Dio et al., 2020). Considering an older population, Vollmer et al. (2018) observed that while adults resist social pressure from a group of small humanoid robots, children aged 7 to 9 conform to the robots’ suggestions. Concerning the age group considered in this study (9–13 years), we conducted a previous experiment where groups of children and teenagers participated in a team strategy game. The robot provided advice that was sometimes correct and sometimes incorrect (Pusceddu et al., 2025). Results show that participants rarely adjust their strategies based on the robot’s advice, especially after it makes mistakes. At the group dynamics level, a potential influence emerges between human players who consistently initiate moves and the rest of the team.

Building on our prior work, we examine robot influence in a spontaneous scenario rather than one requiring strategy. Playful activities have proven to be versatile tools in HRI, as they may be customized to various participant types and used to test different aspects of interaction (Rato et al., 2023; Pasquali et al., 2021; Strohkorb et al., 2016; Pereira et al., 2012; Castellano et al., 2009), while simultaneously reducing the Hawthorne effect, distracting people from the idea that they are experiment subjects and promoting unbiased human behaviors (Chiesa and Hobbs, 2008). For this reason, we developed a dance task designed to capture immediate and natural aspects of group influence, observing whether and how often participants imitate the robot during the game.

Research about imitation on preschool-aged children (1–3 years old) suggests that they imitate human actions more frequently and with greater accuracy than those performed by robots (Sommer et al., 2020; 2021). In older age groups, studies mostly focus on children and teenagers with autism spectrum disorder. Findings suggest that imitation learning from social robots is often more engaging than from human therapists for children with autism (Zheng et al., 2014; 2016). However, results found for this specific population are not easily generalizable to typically developing children. Although it was very valuable to analyze studies about children’s imitation in order to create our experimental design, our work does not focus on imitation performance but uses the phenomenon of imitation to evaluate the influence of a robotic agent within a group setting during a dance game. More specifically, we intend to explore how a robot’s actions shape group dynamics and influence action execution among elementary and early middle school children.

## 3 Materials and methods

### 3.1 Participants

The participants were students involved in an educational project called *NaoToKnow*, which aimed to teach primary and secondary school students how to program Nao and some general concepts about social robotics. Further details about the *NaoToKnow* project can be found in the Acknowledgements section. A subset of the students participating in the above-cited initiative took part in our study after the approval of the Ethical Committee of the University of Genoa (protocol n. 20220317, 03/17/2022). Through the above-cited project, at the moment of the experiments, the participants were familiar with the Nao robot and had a basic understanding of its functionality. We can assume that this familiarity with the robot helped mitigate the novelty effect that is often present in HRI studies, where participants’ behavior may be influenced by initial excitement.

Seventy-two children between nine and 14 years old participated in the study (24 males, 46 females, 2 not declared; age: μ=10.8 , σ=1.6 ). The children participated in the experimental task in teams of four; thus, eighteen groups were tested.

### 3.2 Setup

**FIGURE 2:**
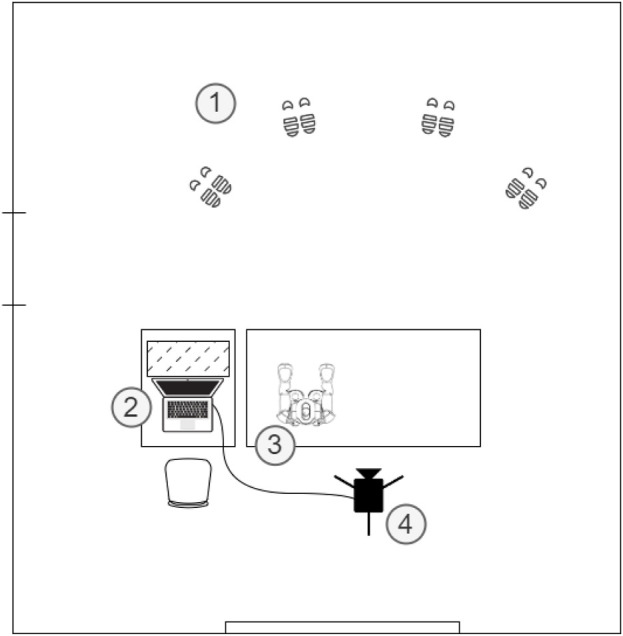
Schema of the setup. (1) Designated positions of the participants; (2) Experimenter position with a laptop positioned behind a separator; (3) Nao on a desk; (4) Webcam with integrated microphone on a tripod.

The study was conducted in a real-world setting: the experimental sessions took place in a classroom of a school in Bracciano, Italy. The Nao robot was placed on a 75-cm-tall desk in the center of the room, facing the participants. Participants stood about 1 m apart one from the other and about 3 m from the robot (see Figures 1, 2 for reference). The designated position of every participant was marked on the floor with tape (1 strip for each foot). A 4K webcam with an integrated microphone to shoot the interaction was installed on a 180-cm tall tripod. A laptop was used to manage the recordings and to store the data temporarily. See Figure 2 for a schema of the setup.

### 3.3 Description of the task

**FIGURE 3:**
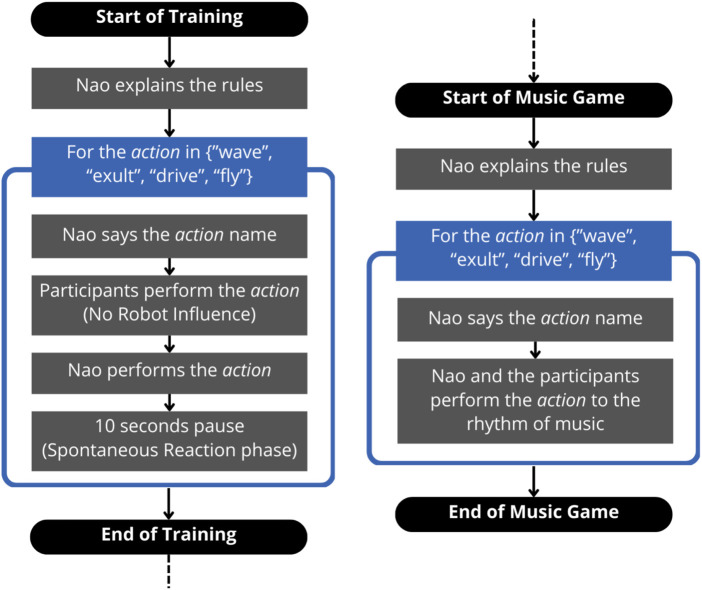
A diagram of the task, divided in Training and Music Game phases.

The activity consisted of a game in which participants and the robot had to mime four actions whose detailed description can be found at the end of this section. The task was in the participants’ mother language (Italian); it lasted about 5 min and comprised two phases. The task is summarized in the diagram in Figure 3, and a video of it is available in the supplementary material.

**FIGURE 4:**
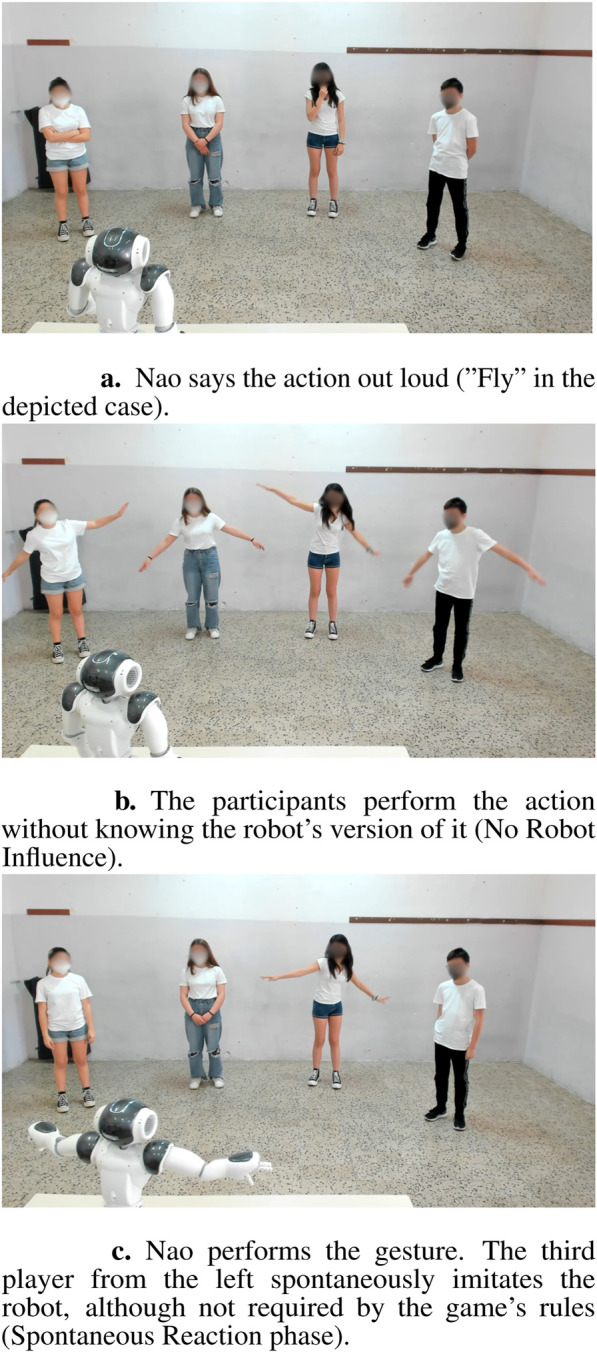
Sequential images illustrating the composition of the Training phase. **(a)** Nao says the action out loud (“Fly” in the depicted case). **(b)** The participants perform the action without knowing the robot's version of it (No Robot Influence). **(c)** Nao performs the gesture. The third player from the left spontaneously imitates the robot, although not required by the game's rules (Spontaneous Reaction phase).

1. **Training**: As the first thing, the robot explained the rules: it would name some actions, and the participants would have to perform them. For every one of the four actions, this Training phase was divided into two steps. In the former, the robot said the action out loud (e.g., “Waving!“) without performing it, and the four participants had to act it out (**No Robot Influence** phase). In the latter, the robot declared that it was going to show its own interpretation of the movement and performed it; after completing the action, Nao remained silent in a resting position for about 10 seconds to allow time for participants to express possible spontaneous reactions to the robot’s gesture before moving on to the next action (**Spontaneous Reaction** phase). See Figure 4 for a visual description of the Training phase.
2. **Music Game**: Nao said: “Now we will repeat all the gestures we have just tried out at the music rhythm” and announced each action out loud; the human participants and the robot executed it together to the rhythm of the *Gioca Jouer* song, popular in Italy.

We designed the experiment with two phases because the Training allowed to understand how the participants perform the actions without the influence of the robot, so that it could be evaluated whether, in the Music Game, after seeing the robot’s actions, they decided to imitate it.

Nao performed two actions in a Typical way and two in an Atypical one. With Typical, we indicated a gesture the robot mimes as an average child does. In contrast, we programmed Nao to perform Atypical actions in an unconventional way, dissimilar to how a person would act. The Typical actions were needed as a baseline to investigate how participants would respond to Atypical actions. This classification was based on the common association between the Gioca Jouer song and its widely recognized dance movements in Italy. We assumed that these gestures reflect a general consensus on how such actions are typically mimed. It was confirmed that the selected gestures were suitable because most participants executed them with a Standard style during the Training phase, even before observing Nao’s version of the gesture (see Section 4 for more details).

The following list contains detailed descriptions of the gestures made by the robot during the experimental tasks in the order of execution:

• Wave (Typical): Nao raises its right arm, places its forearm in a vertical position, and starts to tilt it from left to right and back.

• Exult (Atypical): Nao slowly lifts its arm straight in the air and says the Italian word for “hurray” with a plain tone of voice. If performed in the Standard way, the same action would require quickly lifting both arms and moving them back and forth.

• Drive (Typical): Nao extends its arms horizontally in front of its torso and tilts them as if holding a car’s steering wheel.

• Fly (Atypical): This gesture is programmed to look like Nao is imitating a gliding plane. It opens its arms straight on the shoulders’ line, then bends one knee at a time, resulting in a tilt of its arms. We consider the Standard action as raising and lowering the arms like the wings of a bird.

### 3.4 Questionnaires

After the activity, participants responded to a questionnaire on a tablet through Survey Monkey^1^. They were asked if they had imitated the robot during the task; then, they answered an adapted version of the Group Environment Questionnaire (Carron et al., 1985), a survey created to measure group cohesion, on a five-point Likert scale. An attention-bump item (“Answer 5 to this question to demonstrate your attention”) was added to verify that the participants were focused on the task. In total, 67 answers to the questionnaire were collected, as 5 participants were excluded because of the invalid answer to the attention bump item.

### 3.5 Data analyses

#### 3.5.1 Video annotations

We performed a *post hoc* analysis of the video recordings concerning annotations on objective aspects of participants’ behavior. To ensure a reliable and systematic annotation and minimize the annotators’ mistakes, the observations were independently made by two individuals chosen among the authors. Their notes were then mediated by a third person, following the approach of Waseem and Hovy (2016). In our case, the third party was a social psychologist, familiar with this methodology.

The annotators worked autonomously without influencing each other. The inter-annotator agreement resulted in an 86.46% match. The mismatches in the annotations were analyzed by the social psychologist, who selected what they considered to be the most appropriate answer between the two annotations.

These annotations were made not only to collect data for the analyses in this study but also to have a ground truth to be potentially used in the future to validate automatic video analysis software.

We distinguished among two types of participants’ **action styles**: (i) *Standard*: the participant performs the action in a human-like way, i.e., they conform to Nao in the Typical actions but behave differently from Nao for the Atypical actions; (ii) *Non-standard*: the participant performs the Atypical actions in the same way as Nao in the Atypical actions; the participant performs the Typical action in a non-expected way. Mixed executions, i.e., the participant performs the gesture partially in a Standard way, partially in an Non-standard one, were considered Non-standard. There were no cases in which children performed Non-standard gestures in a manner different from the Atypical gestures shown by NAO.

For every action in both the Training and the Music Game phases, the following annotations were made for each human participant:

1. The action style they used to perform the action;
2. Whether they were the first player to start the action;
3. Whether they spontaneously imitated the robot after it demonstrated its version of the action (in the Spontaneous Reaction phase).

From item (1) of the list, we computed the frequency of possible action styles for every player and group in every task’s phase, to gain an insight about their tendency to conform to the others. Through (2), we could identify which participant was the initiator for every trial and compute the occurrence of starting-as-first times for every player, hence assessing their proactiveness. Item (3) provided instead an indirect measure of the engagement level of the participant, who spontaneously decides to imitate the robot.

#### 3.5.2 Strong proactive player groups

From the observation of group behaviors, we noticed that some groups presented a player keener on starting the action without waiting for the others. We hypothesized that the presence of such a player could drive some dynamics of the whole group and somehow influence the decision to imitate the robot.

We then formulated a criterion to distinguish these groups from the others. We only considered the rounds of Atypical gestures because they were the trials in which it was more evident whether the players conformed to other members, including the robot. We identified as *Strong Proactive Player* (SPP) *groups* the teams in which one member initiated the gesture first at least 75% of the time, of which at least one solo. Sometimes more than one player started the action together.

## 4 Results

### 4.1 Q1: do participants conform to a robot’s atypical behavior during a social game?

#### 4.1.1 Participants’ overall imitation of the robot

The results of the survey reveal that, when the participants were asked whether they imitated the robot during the Music Game, 69.8% of them answered “yes”, 22.2% “sometimes”, and 7.9% “no”. According to the annotations, this is in line with participants’ actual behavior for the Standard actions in the Music Game phase, in which they acted as the robot in 98.6%. However, in only 43.8% of the Atypical trials, in the Music Game phase, participants performed the Atypical actions in a Non-standard way (i.e., in the same way or partially as Nao).

#### 4.1.2 Differences in behavior between training and music game

In the Training phase, before seeing Nao’s version of the actions, children mostly performed the gestures in a Standard way (88.2%) with respect to a Non-standard style (11.8%), thus validating our design choices for Nao’s execution of the Typical and Atypical actions. Additionally, if participants perform the gestures in a Standard manner before observing the robot’s actions, it demonstrates that the robot’s Atypical actions are distinct from their usual behavior, thereby justifying the selection of these Atypical actions.

**FIGURE 5:**
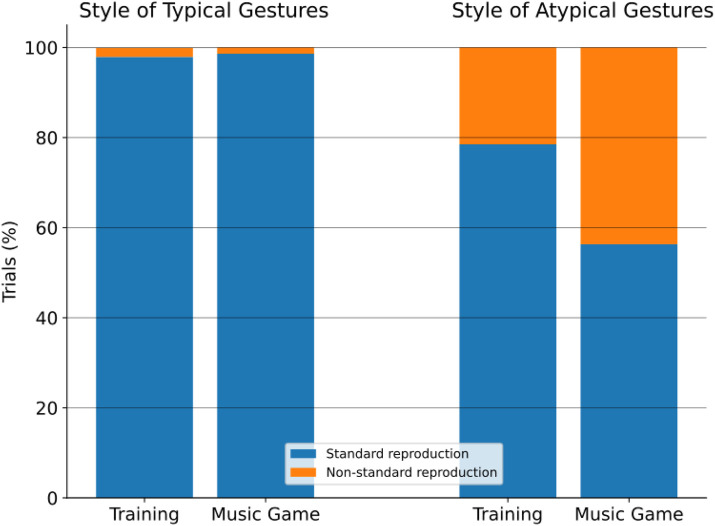
Comparison of players’ performance of Typical and Atypical gesture styles before (Training) and after (Music Game) exposure to Nao’s version. The plot highlights shifts in gesture style preferences before and after exposure to the robot’s action style, suggesting potential influence on participants’ behavior.

Significant differences between the Training (No Robot Influence) and Music Game phases (after having seen Nao’s action) were found for the Atypical gestures, where the Non-standard executions of the participants doubled in the latter phase, with respect to the Training (Non-standard executions in Training: 21.5%, in Music Game: 43.8%. McNemar’s tests: p<.001 for “Fly” and p=.005 for “Exult”). The increase in the execution of actions in a Non-standard manner suggests that participants imitated Nao’s Atypical actions in the Music Game. No before-after difference was observed for distributions regarding the Typical actions, as the percentage of Non-standard executions was 2.1% in Training and 1.4% in Music Game. These results are summarized in Figure 5.

#### 4.1.3 Spontaneous imitation

In the Spontaneous Reaction phase, participants naturally mimicked the robot in 35.4% of the trials, even though it was not part of the game’s rules. With 47.2% of trials, “drive” (Standard) was the most spontaneously imitated gesture, followed by “wave” (40.2%, Standard), “fly” (29.2%, Atypical), and “exult” (25.0%, Atypical). A Binomial Logistic Regression was conducted to verify if imitating the robot in the Spontaneous Imitation phase could predict the behavior in the Music Game phase for the Atypical actions, but the test did not produce any significant statistical results (p=.072) .

### 4.2 Q2: are the participants influenced by the other group members during a social game with a robot?

#### 4.2.1 Single instance behavior

**FIGURE 6:**
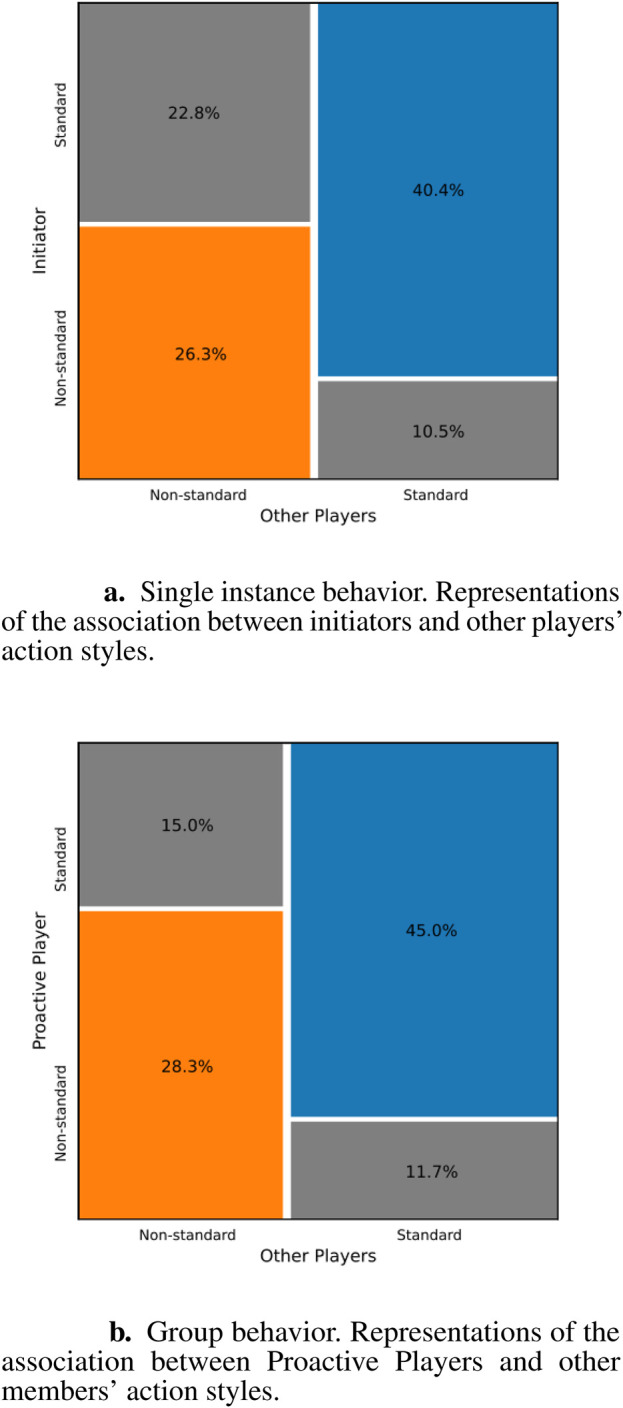
Mosaic representations of the contingency tables illustrating the association between initiators/Proactive Players and other players’ action styles. The width of each segment corresponds to the relative frequency of occurrences. Chi-square tests results indicate a significant relationship between initiator/Proactive Player and other players’ actions, suggesting a potential influence of the former on subsequent players’ actions. **(a)** Single instance behavior. Representations of the association between initiators and other players' action styles. **(b)** Group behavior. Representations of the association between Proactive Players and other members’ action styles.

We investigated whether, in trials where the robot performs the Atypical action (2 Atypical rounds per group, hence 36 Atypical rounds in total), players’ execution could be mediated by the presence of an initiator (i.e., a player who started the action before the other human participants). To do so, we analyzed every trial of the Atypical gestures (“Fly” and “Exult”) in the Music Game phase in which there was an initiator. These were 19 out of 36 rounds; in the remaining trials, more than one player started the action simultaneously. Since there are three potential “imitators” in each round, we conducted these analyses on a sample size of N=57 . In this condition, we checked whether the group members made the action with the same style as the initiator. The contingency table - represented in Figure 6a - was tested with a Chi-square test, that resulted significant ( $\chi^{2}=6.62$ , p=.010 ), meaning that there is a relation between the initiator’s and other players’ action style. The Chi-square test does not reveal causality effects, but in our case, the initiator starts the action before the other players, then we can hypothesize that the initiator influences the other players, while *vice versa* is not possible. Even with this temporal sequence, establishing causality remains challenging. While it is intuitive to think that the initiator’s action might influence others, there could be other group dynamics factors at play.

**FIGURE 7:**
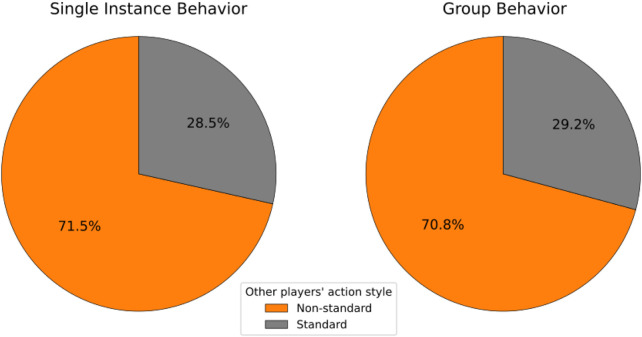
Other players’ action style when initiator/Proactive Player’s style is Non-standard.

In light of these results, further considerations can be made regarding the imitation of Nao by the participants. In Figure 6a, it can be observed that, among the 36.8% of cases in which the initiator performs a Non-standard action, the other players match the Non-standard execution the 26.3% of the time, relative to these cases (as indicated by the orange box), with the remaining 10.5% where the other players perform the action in a Standard way (grey box, bottom right). An additional representation of these data can be found in the pie chart to the left of Figure 7: when the initiator makes a Non-standard reproduction of an Atypical action, 71.5% of the other members do the same. It can therefore be stated that players perform the action like the robot more frequently if the initiator has done the same. The opposite is also true: when the initiator decides not to imitate the robot, the other players do the same (blue box).

#### 4.2.2 Group behavior

The groups of participants attended the same school class. We could not know in detail the dynamics of pre-existing relationships among group members, but to acquire a general idea of their cohesiveness, we ran Group Environment Questionnaires. A One-Sample t-test against the neutral value (3, on a 1-5 Likert scale) revealed that, on average, participants perceive their team as cohesive (μ=4.2±0.4,p<.001) .

According to the group categorization presented in the Analyses, we identified 10 Strong Proactive Player (SPP) groups out of 18. For each SPP group, we identified one Proactive Player (see Section 3.5.2 for further details).

Similarly to the Single Instance Behavior analyses, we focused on the SPP groups during Nao’s execution of Atypical actions in the Music Game phase (10 SPP groups with 3 non-Proactive Players, 2 rounds, hence N=60 ). We then built a contingency table containing the distribution of action styles between the Proactive Players and the others. The table - whose graphical representation can be found in Figure 6b - categorizes the trials with separate counts for each style (Standard, Non-standard). A Chi-square test was run on the contingency table. It resulted significant ( $\chi^{2}=12.3$ , p<.001 ), evidencing that there is an association between the Proactive Player’s and other players’ action style. Again, we can hypothesize that it is the action style of the proactive player influencing other members, because the Proactive Player starts the action before the regular members, although further data is needed to validate this hypothesis, as there could be other reasons for this phenomenon, such as participants using the gestures they are used to rather than being influenced by the Proactive Player.

After evaluating the affinity between the Proactive Player and the other players, we now assess the execution of the participants’ action styles in relation to that of the robot. The results presented in Figure 6b show that when the Proactive Player decides not to imitate Nao (60.0% of the instances), the other players do the same in the majority of cases (45.0%, blue box), and less frequently decide to imitate the robot anyway (15.0%, grey box at the top left). From the same figure, it can be seen that when the Proactive Player conforms to Nao (40.0%), players tend to imitate the robot’s actions more frequently (28.3% out of 40%, orange box), and perform a Standard reproduction less frequently (11.7% out of 40.0%, bottom right grey box); indeed, the orange box is larger than the grey box at the bottom right. For clarity, this data is also presented in Figure 7, which shows that when the Proactive Player performs an Atypical action like the robot (Non-standard reproduction), the other players do the same in 70.8% of cases.

## 5 Discussion

This work investigates the influence exerted by human peers and the robot Nao on the behavior of the group members during a motor imitation game. In particular, (1) we explored the influence of the robot on group members, investigating whether participants conform to the unconventional behavior of a robot during a social game; (2) we then studied if the influence of the robot on group members might be mediated by human peers.

The study was conducted in a natural school setting rather than a controlled laboratory environment. This approach offers the advantage of observing children in a familiar context, likely leading to more authentic behavior even though it makes obtaining precise measurements more difficult.

In the questionnaire, most of the participants declared to have imitated the robot during the game, but they did not entirely. On one side, the children might feel they have imitated because, in the Standard cases, they performed the action like the robot. However, a disparity was highlighted between the self-reported measures and the actual observation of the Atypical actions. In the questionnaire, participants declared to have imitated the robot more with respect to the video observations. We hypothesize that participants might not want to disclose their lack of willingness to imitate the robot, suggesting they chose the answer they judged as “right”. This might be due to social desirability, i.e., the tendency for individuals to respond in culturally sanctioned ways (Stöber, 2001; Grimm, 2010). We also hypothesize that this behavior might be attributed to the experiments taking place in a school where students are continuously evaluated. Another possible explanation for this result is that participants did not perceive Nao’s Atypical actions as different or interpreted them merely as mistakes by the robot. As a result, they may have been following what they believed the robot intended to do rather than what it actually did. Even though they did not imitate Nao as much as they declared, some showed engagement and curiosity towards the robot as demonstrated by their propensity to imitate it spontaneously; indeed, in the Spontaneous Reaction phase, they imitated the robot’s actions even when it was not required by the game’s rules.

In the vast majority of the trials, participants performed the actions with a Standard style in the Training phase, before seeing Nao’s version. It was then verified that the chosen gestures provided a solid baseline to evaluate Typical and Atypical action styles.

It was unexpected that, despite the robot being positioned in a leadership-like role—placed on the teacher’s desk (“head-of-the-table effect” (Forsyth, 2018)), dictating rules, and guiding the interaction—participants imitated its movements in fewer than half of the trials. These findings might suggest that young people are unwilling to follow a robotic leader if they perceive its indications as unusual or out of their comfort zone. Another reason for this behavior could be that, even though we intended to put Nao in a leadership position, participants did not perceive it as such, similar to a previous study Alves-Oliveira et al. (2016), in which students evaluated Nao as a peer, even though it was acting as a teacher. Another possible explanation for this behavior, similar to the one mentioned above regarding the discrepancy between actual and self-reported measures, is that children may not have fully noticed the deviation from the Typical actions or may have perceived it simply as a mistake by Nao. As a result, they might have been following what they believed Nao intended to do rather than what it actually did. Future studies should investigate this by, for example, asking children whether they noticed that Nao performed the actions incorrectly and why they believed this to be the case. An additional interpretation of why participants rarely performed atypical actions is that they might have felt uncomfortable doing so, as such movements might contrast with group social norms and could elicit ostracism (Forsyth, 2018). This feeling could be heightened if group members perceive their group as heterogeneous since exclusionary reactions are more likely in such cases (Gerard, 1953). However, given that participants reported a high level of group cohesion in the Group Environment Questionnaire, we can reasonably exclude heterogeneity as a contributing factor. Future studies should incorporate a scale to measure social desirability to determine whether this factor could be correlated to the observed behavior (Stöber, 2001).

Results show that initiators influenced others’ behaviors. We found that players made the same action as the robot significantly more often when the initiator conformed to Nao in comparison to when they did not. The same effect was found considering the SPP groups only, referring to a single Proactive Player for each group in place of one initiator for each trial. These findings might indicate that the initiator’s behavior influences the tendency of the other members to conform to Nao and that, in front of the unconventional behavior of a social robot, young participants prefer to imitate a human peer rather than conform to the robotic member of the group. Interestingly, similar results were found in our previous study in a strategy game scenario (Pusceddu et al., 2025), thus suggesting that taking the initiative is a leadership trait (King, 2010; Yoo and Alavi, 2004), despite of the task type.

This observation is relevant for HRI because the acceptance and interaction with a robotic agent, which can be challenging for shy or distrustful children or teenagers, could be enhanced by inserting them in groups with peers who are more willing and predisposed to accept or follow the robot. This could also be reflected in educational contexts. If a robot tries to guide children through unfamiliar concepts or atypical behaviors, it might not be listened to by the students; this would limit its effectiveness as an educational tool. However, a robot tutor aligning with the tendencies of proactive students could enhance engagement, making it a more effective tool for promoting learning. These results could also be relevant in the field of children’s safety. The findings suggest that children and teenagers rarely follow a robot that displays atypical behavior. However, they tend to follow proactive members of the group, which could pose a risk if those proactive members were to rely on a robot with unusual behavior without questioning it. Thus, these findings open up possibilities for leveraging robots’ influence in groups for children’s benefit, but also raise concerns about how this influence could have negative effects.

To investigate this effect deeper, in future studies, it would be useful to observe how participants would behave in the presence of a proactive player acting atypically and a robot acting in a standard way. Furthermore, groups with a Strong Proactive Player might have other peculiarities worth studying. For example, the presence of a proactive player might impact the group’s cohesion. From another perspective, in groups without proactive human members, a robot exhibiting proactive qualities could play a valuable role in improving team performance and unity.

In general, dividing groups into categories might help better understand their different strengths and needs. Based on this study, a possible criterion for categorization could be the presence or absence of proactive group members. This kind of classification could allow researchers to design and personalize robots to better align with the dynamics of each group to help improve performance and enjoyment.

This study also presents some limitations. For instance, the actions pool was limited to only four, due to time constraints. Testing a wider variety of actions would be of interest and could provide stronger conclusions. In addition, as the groups were not perfectly homogeneous with respect to age and gender and because the sample size was limited, it was not possible to perform reliable analyses to test for any effects of the subjects’ age and gender. As a future improvement, the manual annotation analysis could be substituted with automatic computer vision software, using the annotations of this study as ground truth to test the algorithms. Automating the annotations would not only simplify and accelerate the analyses for researchers but also open the possibility for real-time behavioral analysis. This advancement could enable robots to dynamically monitor group behaviors, including those of proactive participants, and adapt their actions accordingly.

In conclusion, this study explores how, in playful contexts with children and teenagers, it is not enough to assign a leadership role to the robot to ensure children’s compliance with the robotic partner: group dynamics must be considered. Despite some limitations, our results add a new dimension to this research, showing that the presence of proactive group members can influence their peers, potentially enhancing or undermining the group’s conformity to the robot.

## Data Availability

The raw data supporting the conclusions of this article will be made available by the authors, without undue reservation.

## Footnotes

1
https://surveymonkey.com
